# Did expanded access to denture services improve chewing ability in the Korean older population? Results of a regression discontinuity analysis

**DOI:** 10.1038/s41598-020-68189-7

**Published:** 2020-07-17

**Authors:** Nam-Hee Kim, Ichiro Kawachi

**Affiliations:** 1 Department of Dental Hygiene, Yonsei University Wonju College of Medicine, Wonju, Republic of Korea; 2 Department of Social and Behavioral Sciences, Harvard T.H. Chan School of Public Health, Boston, MA USA

**Keywords:** Health care, Medical research

## Abstract

The Korean National Health Insurance expanded the dental insurance in 2012 to cover denture services for older adults. We analyzed whether the new policy improved of chewing ability in the eligible population. We used regression discontinuity (RD), a quasi-experimental design, to analyze the effects of the expanded dental insurance. We analyzed data from the Korea National Health and Nutrition Examination Survey conducted in 2010 and 2015. The study population consisted of two groups: the treatment group, aged 65 and above who were eligible; and the control group, under 65 years of age who were not eligible for the dental insurance benefit. The main outcome evaluated was self-reported chewing difficulty. The RD analysis showed that in 2015, the chewing difficulty in aged above 65 was 2.2% lower than in those aged under 65. However, the difference was not statistically significant (P = 0.76). The results from the falsification testing of predetermined covariates, placebo cut-offs, and bandwidths validated our main conclusion. The expansion of dental insurance benefits to include dentures for the older adults did not improve the chewing ability in the eligible population. Future studies should evaluate long-term outcomes of oral health as well as the social impacts on the elderly.

## Introduction

An important global goal related to oral health, as set forth by the World Health Organization (WHO), is the reduction of chewing difficulties in the older adults^1^. Consequently, national strategies have been launched to achieve this goal. In 2012, the Korean government expanded dental insurance for the older adults to include coverage for removable complete and partial dentures. Furthermore, in 2014, dental implant services were included in the National Health Insurance scheme. The expansion of insurance coverage for geriatric dental care ensures the well-being of an aging society and specifically aims to improve oral health outcomes for the older adults.

Discomfort when eating food, which is measured as chewing difficulty, is the most frequently reported dental issue in the older adults^1–4^. Denture treatment is a common rehabilitation procedure used to enhance chewing ability in the older adults, in order to improve nutrition intake and quality of life^3–5^. In Korea and other developed countries, as much as one-third to a half of all elderly people wear complete dentures, and up to three-quarters wear removable complete or partial dentures^6^. We expected that there is increasing interest globally in assessing the impact of dental insurance coverage for dentures on oral health.

However, in contrast to studies on medical insurance, there is limited evidence on the effect of dental insurance on oral health. This may be a reflection of the lower priority accorded to dental care compared to medical care, which is indicated by the late introduction of dental insurance, fewer benefits, and limited coverage, all of which leads to out-of-pocket expenditure for beneficiaries^7,8^. In turn, due to these limitations, there is a lack of evidence supporting the benefits of a dental insurance policy.

Reports on dental insurance focus on the need for coverage, implementation, disparities in accessibility, and inequalities^7,9–14^. Cost reduction is also one of the main foci of dental insurance studies^7,8,15,16^. For example, within the Korean context, after the expansion of dental insurance, the accessibility of dental care improved^10,11,14^. However, there have been inconsistent findings on the impact for oral health disparities, with some reports indicating a reduction^12^, while others reporting widening inequalities^9,13,14^. However, causal inference has been hampered by the paucity of studies featuring a strong identification strategy^17–19^.

In the USA, reports using a difference-in-difference design showed that insurance expansion under the Affordable Care Act of 2010 increased dental visits^20^ but did not reduce income-based inequalities in oral health^21^. A single report focused on the elderly and used a regression discontinuity (RD) design to evaluate the impact of cost sharing on the use of dentures^15^. Even though the utilization of dentures increased, no improvement in chewing ability was found. Thus, more studies are required to assess the impact of insurance coverage for denture services.

To establish a causal inference, randomized trials are widely considered to be the gold standard^18,19^. Although an observational study may identify an association between denture treatment and improved chewing ability, it may not necessarily imply the cause and effect because of unobserved patient characteristics, which may be correlated with both demand for dentures and oral health outcomes^17–19^. For example, patients with high oral health literacy might be more likely to use denture services. Such patients are also likely to have better oral health outcomes regardless of whether they use dentures. Therefore, a comparison of denture users and non-users will be biased.

Previously, randomized controlled trials (RCTs) have shown that denture treatment increases bite force and chewing ability in geriatric patients^22,23^. However, these results may not always translate into improved oral health outcomes in the real world^18,19^. Therefore, a rigorous evaluation of policy is also required. That is, RCTs can establish the efficacy of treatment, but effectiveness is established by policy evaluation^24^.

We therefore aimed to evaluate the policy adopted in 2012 by the Korean government to expand access to dentures for the older adults. We adopted an econometric RD design, which is a quasi-experimental approach that identifies the causal effects of an intervention using a cut-off or threshold (in this instance, the age of eligibility to receive denture insurance benefits) to assign the intervention. Despite the absence of an experimental design, an RD design can exploit exogenous characteristics of the intervention to identify causal effects. If all individuals above the cut-off age are eligible for the insurance benefit, it is possible to identify the local treatment effect by comparing individuals just above to those just below the cut-off age^19,25^. It is expected that an individual aged 64 years is likely to be similar in terms of chewing ability to someone aged 65 years. However, the former will not be eligible for insurance coverage for dentures, whereas the latter will be eligible. Comparing the outcome of the eligible group to the counterfactual outcome of the non-recipient or control group can help identify the local treatment effect.

## Materials and methods

### Study design and population

We used a regression discontinuity, quasi-experimental study design. We analyzed data from the KNHANES for 2010 and 2015, a nationally-representative, cross-sectional survey conducted by the KCDC. We restricted our study population to adults aged between 50 and 80 years to maintain the same number of observations for analysis in each year. To perform a post-treatment comparison in the RD before the policy (2010) and after the policy was implemented (2015), we included 1,634 (< 65 years) and 1,230 (≥ 65 years) respondents and 1,361 (< 65 years) and 1,178 (≥ 65 years) respondents, respectively.

### Study variables

The main outcome studied in the RD analysis was chewing difficulty; the running variable was age, for which the assigned cut-off was 65 years: aged under 65 years (not eligible for dental insurance benefits) and aged 65 years and over (eligible for dental insurance benefits). Chewing difficulty was assessed using self-responses to the following in-person questions: ‘Do you have difficulty or discomfort when chewing food due to oral problems, including teeth, dentures, or gums?’ and ‘If you use dentures, please describe your experience of wearing dentures’. Individual responses of ‘no difficulty’, ‘little difficulty’, and ‘some difficulty’ were categorized as ‘no chewing difficulties’ and assigned a value of 0; whereas responses of ‘difficulty’ and ‘severe difficulty’ were categorized as ‘chewing difficulties’ and assigned a value of 1. Fourteen variables were included in our RD model as predetermined covariates, namely gender (men/women), education (elementary school/middle school/high school/university), income (low/low-middle/middle-high/high), spouse (with/without), self-reported oral health (good/poor), oral examination (yes/no), dental visit (yes/no), unmet dental needs (yes/no), self-reported general health (good/poor), unmet medical needs (yes/no), denture wearing status (yes/no), denture needs (yes/no), no. of remaining teeth (excluding wisdom teeth) and presence of more than 20 teeth (yes/no).

The reference period for these questions, excluding gender, education, and income status was the previous year. Oral examination and dental visit were assessed by whether the individual reported receiving an oral examination (dental visit) in the previous year. Unmet dental (medical) needs was evaluated based on whether the respondent reported that they needed dental (medical) care but was unable to receive treatment within the past year. Poor self-reported oral (general) health was defined as those who reported poor (and/or very poor) oral (general) health status from a single item that included five response options: very good, good, moderate, poor, and very poor. Denture status presented that there was one or more had which of partial and/or complete dentures on the upper and/or lower status. Denture needs was measured that there were one or more needs of denture treatment which of partial and/or complete dentures on the upper and/or lower by the dentist. Denture status, denture needs, number of remaining teeth and the presence of more than 20 remaining teeth were assessed by oral examination according to the WHO guidelines^26^.

### Statistical analysis

The analysis was based on the canonical sharp RD design in which individuals aged 65 years and above were assigned to the treatment condition, and those aged 64 years and under were assigned to the untreated or control condition. The RD cut-off of 65 years was used to ensure that all units were treatment compliant^27,28^.

First, we evaluated the RD treatment effect using robust bias-corrected inference with mean-squared error (MSE)-optimal and coverage error (CER)-optimal bandwidth. For this phase, we graphically represented RD plots of chewing difficulty to: (i) demonstrate features of the RD design using survey data from 2010 and 2015 and (ii) compare the changes in the RD plots of data before versus after the policy change. All RD plots considered the same duration within each treatment assignment status with the MV (mimicking variance) and ES (evenly spaced) bins. To assess variability of chewing difficulty, we used the selected bins to illustrate plots with the integrated mean-squared error (IMSE)-optimal bandwidth.

Next, we investigated crude and covariate-adjusted RD treatment effects using MSE-optimal and CER-optimal bandwidth with covariates. The covariate-adjusted estimation included all predetermined covariates that fit a weighted least squares regression of chewing difficulty for data in 2010 and 2015, which was done using the ‘rdrobust’ command and the ‘covs’ option.

Finally, we verified the validation and falsification of the RD design for data from 2015 in the following four stages: (a) the null treatment effect on pre-treatment covariates, (b) continuity of the score density around the cut-off, (c) the treatment effect at artificial cut-off values, and (d) the sensitivity to bandwidth choices. We repeated these analyses for all the covariates to provide a complete falsification test using the same estimation and inference procedures.

- We created an RD plot for each of the ten covariates with mimicking variance and evenly-spaced bins. Since the predetermined covariates could not have been affected by the treatment, the null hypothesis that there would be no treatment effect could not be rejected if the RD design was valid. We estimated the local linear RD effect using triangular kernel weights, MSE-optimal bandwidth, and CER-optimal bandwidth for each covariate.
- We used the density test to graphically represent the density of the running variable (age). We selected a small neighborhood around the cut-off and performed a simple Bernoulli test for the area with a 50% probability. The null hypothesis was that there was no manipulation of density at the cut-off.
- We conducted the falsification test for the cut-off (age 65 years) by determining whether there were significant effects at the artificial or placebo cut-off values. These values differed from the true cut-off value by ± 3 years. Therefore, those aged 65 years and above did not markedly change. We expected that there would be no significant treatment effect with placebo cut-off values. The analysis was performed using local-polynomial methods within an optimally-chosen bandwidth around the artificial cut-off to estimate treatment effects on chewing difficulty.
- We also tested the sensitivity of the results to bandwidth choice, which investigates sensitivity while units from the center of the neighborhood around the cut-off are removed. The analysis was performed using local polynomial methods with different bandwidths (3.174–6.348 and 4.694–9.388), to assess if the bandwidth affected the final results.

We used the Stata statistical software (Stata Corp. 2017. Stata Statistical Software: Release 15. College Station, TX: Stata Corp LLC.) for all statistical analyses. All differences were statistically significant with a P < 0.05.

### Ethical approval and informed consent

This study used open access data from the Korea National Health and Nutrition Examination Survey (KNHANES) for 2010 and 2015 conducted by the Korea Centers for Disease Control and Prevention (KCDC). All participants of the KNHANES agreed the informed consent to participate in the survey. The KNHANES was approved by the Institutional Review Board (IRB) of the KCDC. The approval numbers of the surveys are 2010-02CON-21-C, and 2015-01-02-6C.

This is a publicly available, secondary dataset. Our institute determined that the use of the KNHANES dataset does not meet the criteria for human subject research, and was therefore exempt from IRB approval. We confirmed that all methods were performed in accordance with the relevant guidelines and regulations.

## Results

### Prevalence of chewing difficulties and study population distribution according to predetermined covariates

The difference between the prevalence of chewing difficulties among those aged 65 years and above and those aged under 65 years was significant across all covariates (P < 0.001) except for gender, for data in 2010 and 2015 (Table 1).

**Table 1:** Proportion of study population and prevalence of chewing difficulty.

Variable	Study population						Chewing difficulty					
	Year 2010			Year 2015			Year 2010			Year 2015		
	< 65	65 ≤	P*	< 65	65 ≤	P*	< 65	65 ≤	P*	< 65	65 ≤	P*
**Total**												
**N**	1,634	1,230		1,361	1,178		558	504		359	516	
**%**	57.0	43.0		53.6	46.4		34.2	41.0		26.5	43.9	
**Sex**												
Male	50.0	42.0	0.085	48.0	43.0	0.181	39.0	41.0	0.161	28.0	40.0	0.982
Female	50.0	58.0		52.0	57.0		33.0	44.0		26.0	45.0	
**Education**												
Elementary	35.0	72.0	< 0.0001	21.0	62.0	< 0.0001	43.0	46.0	< 0.0001	38.0	51.0	< 0.0001
Middle	24.0	11.0		20.0	12.0		36.0	37.0		28.0	34.0	
High	29.0	11.0		33.0	18.0		33.0	30.0		24.0	30.0	
University	13.0	6.0		26.0	8.0		24.0	40.0		19.0	27.0	
**Income**												
Low	16.0	53.0	< 0.0001	14.0	45.0	< 0.0001	48.0	47.0	< 0.0001	36.0	52.0	< 0.0001
Middle-low	27.0	23.0		24.0	28.0		36.0	37.0		31.0	40.0	
Middle-high	27.0	13.0		26.0	18.0		37.0	38.0		22.0	36.0	
High	30.0	10.0		36.0	9.0		30.0	39.0		24.0	30.0	
**Spouse**												
Without	12.0	34.0	< 0.0001	14.0	36.0	< 0.0001	41.0	44.0	< 0.0001	29.0	49.0	< 0.0001
With	88.0	66.0		86.0	64.0		35.0	42.0		26.0	40.0	
**Oral health**												
Good	47.0	42.0	0.018	49.0	46.0	0.105	17.0	21.0	< 0.0001	9.0	26.0	< 0.0001
Poor	53.0	58.0		51.0	54.0		53.0	59.0		44.0	58.0	
**Oral examination**												
No	79.0	86.0	< 0.0001	63.0	77.0	< 0.0001	37.0	41.0	0.004	27.0	46.0	0.002
Yes	21.0	14.0		37.0	23.0		32.0	50.0		27.0	33.0	
**Dental visit**												
No	92.0	94.0	0.043	40.0	46.0	0.070	35.0	43.0	0.004	23.0	40.0	0.002
Yes	8.0	6.0		60.0	54.0		44.0	42.0		30.0	46.0	
**Unmet dental needs**												
No	59.0	72.0	< 0.0001	69.0	72.0	0.011	24.0	37.0	< 0.0001	21.0	35.0	< 0.0001
Yes	41.0	28.0		31.0	28.0		54.0	57.0		40.0	65.0	
**General health**												
Good	76.0	64.0	< 0.0001	81.0	69.0	< 0.0001	33.0	38.0	< 0.0001	24.0	37.0	< 0.0001
Poor	24.0	36.0		19.0	31.0		46.0	51.0		39.0	57.0	
**Unmet medical needs**												
No	80.0	79.0	0.325	88.0	88.0	0.612	33.0	40.0	< 0.0001	25.0	40.0	< 0.0001
Yes	20.0	21.0		12.0	12.0		47.0	53.0		41.0	64.0	
**Denture wearing**												
No	46.0	19.0	< 0.0001	36.0	16.0	< 0.0001	29.5	36.0	0.025	22.2	37.6	0.031
Yes	54.0	81.0		64.0	84.0		37.7	43.3		31.2	45.0	
**Denture needs**												
No	84.0	78.0	< 0.0001	68.0	63.0	< 0.0001	29.4	37.4	< 0.0001	33.7	40.9	0.003
Yes	16.0	22.0		32.0	37.0		64.7	57.2		40.9	49.4	
**Presence of more than 20 teeth**												
No	17.0	54.0	< 0.0001	11.9	40.1	< 0.0001	54.8	49.9	< 0.0001	47.3	53.9	< 0.0001
Yes	83.0	46.0		88.1	59.9		29.7	32.8		25.0	36.5	
**No. of remaining teeth**												
Mean (se)**	23.7 (0.14)	16.9 (0.25)	< 0.0001	24.6 (0.13)	18.7 (0.24)	< 0.0001	21.5 (0.29)	14.4 (0.37)	< 0.0001	22.8 (0.29)	16.7 (0.38)	< 0.0001

*P-value for the Chi-square test and t-test, when comparing the two groups.

**Denotes survey mean and standard error.

The distributions of the study populations are shown in Table 1. Except for gender, there were significant differences in the socioeconomic status between the two groups for data in 2010 and 2015. In general, those aged 65 years and above were less likely to be educated and more likely to report a lower income. With regard to clinical oral health status, individuals aged over 65 years had more likely wore a denture, fewer teeth, and were less likely to have more than 20 remaining teeth. In addition, they reported that their oral as well as general health was poorer in 2010 and 2015 than the control. However, in 2015, there were no significant differences in self-reported oral health between the two groups, whereas self-reported general health was worse in the older group (P < 0.001). There were no significant differences in dental visits, whereas individuals over the age of 65 years had fewer oral examinations than the control (P < 0.001; Table 1).

### RD plot for chewing difficulties

**Figure 1:**
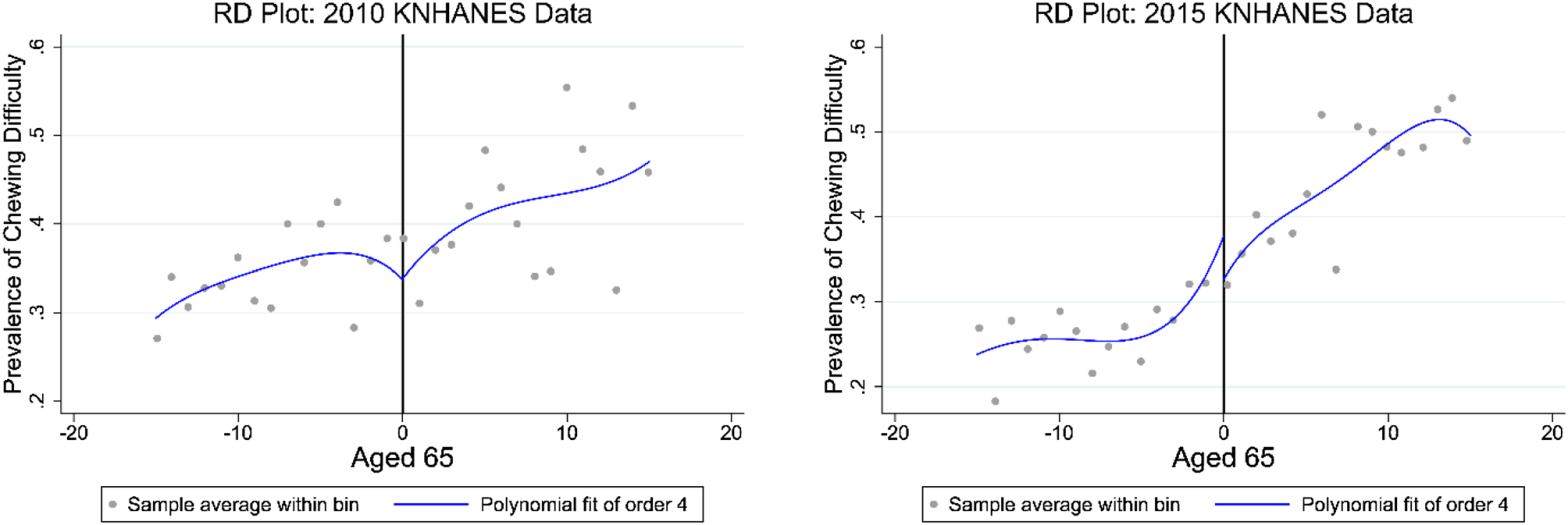
RD plots of chewing difficulty on ‘aged 65’ using survey data from 2010 and 2015.

The RD plots for chewing difficulties are shown in Fig. 1. Chewing difficulty above the cut-off (aged 65 years) showed that there were no discontinuities in 2010, while there were discontinuities in 2015. The RD plots illustrated the vertical distance (discontinuity) between the regression curves for the two groups. It was assumed that the values of the average potential outcomes at ‘aged 65’ did not abruptly vary from the values at points near the cut-off.

### RD effect of chewing difficulty

Table 2 shows the RD point estimates and inference for the chewing difficulty in the two groups. For data from 2015, the adjusted estimated RD effect ranged from − 2.2 to − 6.2% compared with the unadjusted estimate of − 1.50 to − 2.04% when using MSE-optimal and CER-optimal bandwidth, respectively. However, we failed to reject the null hypothesis, which postulated that there would be no difference between the two groups at the cut-off. Nevertheless, this similarity was reassuring because the covariates were truly predetermined: the unadjusted estimator and the covariate-adjusted estimator represented the same parameter and should have resulted in similar estimates^29^.

**Table 2:** Continuity-based approach for RD analysis for chewing difficulty in 2015.

Variable	RD estimator	Robust inference			BW est. (h)	BW bias (b)	Rho (h/b)	Eff. no. of OBS	
		P-value	CI					Left	Right
MSE-optimal bandwidth^a^	− 0.015	0.80	− 0.21	0.17	4.69	8.09	0.58	329	416
CER-optimal bandwidth^a^	− 0.020	0.82	− 0.25	0.19	3.17	8.09	0.39	250	345
MSE-optimal bandwidth with covariates^b^	0.022	0.76	− 0.24	0.17	3.76	6.16	0.61	250	345
CER-optimal bandwidth with covariates^b^	− 0.062	0.60	− 0.32	0.18	2.54	6.16	0.41	171	250

^a^Sharp RD estimates using local polynomial regression.

^b^Covariate-adjusted sharp RD estimates using local polynomial regression. Covariates included gender, education, income, spouse, oral health, oral examination, dental visit, unmet dental needs, general health, unmet medical needs, denture wear status, denture deeds, number of remaining teeth and presence of more than 20 teeth.

### Validation and falsification for RD design

**Figure 2:**
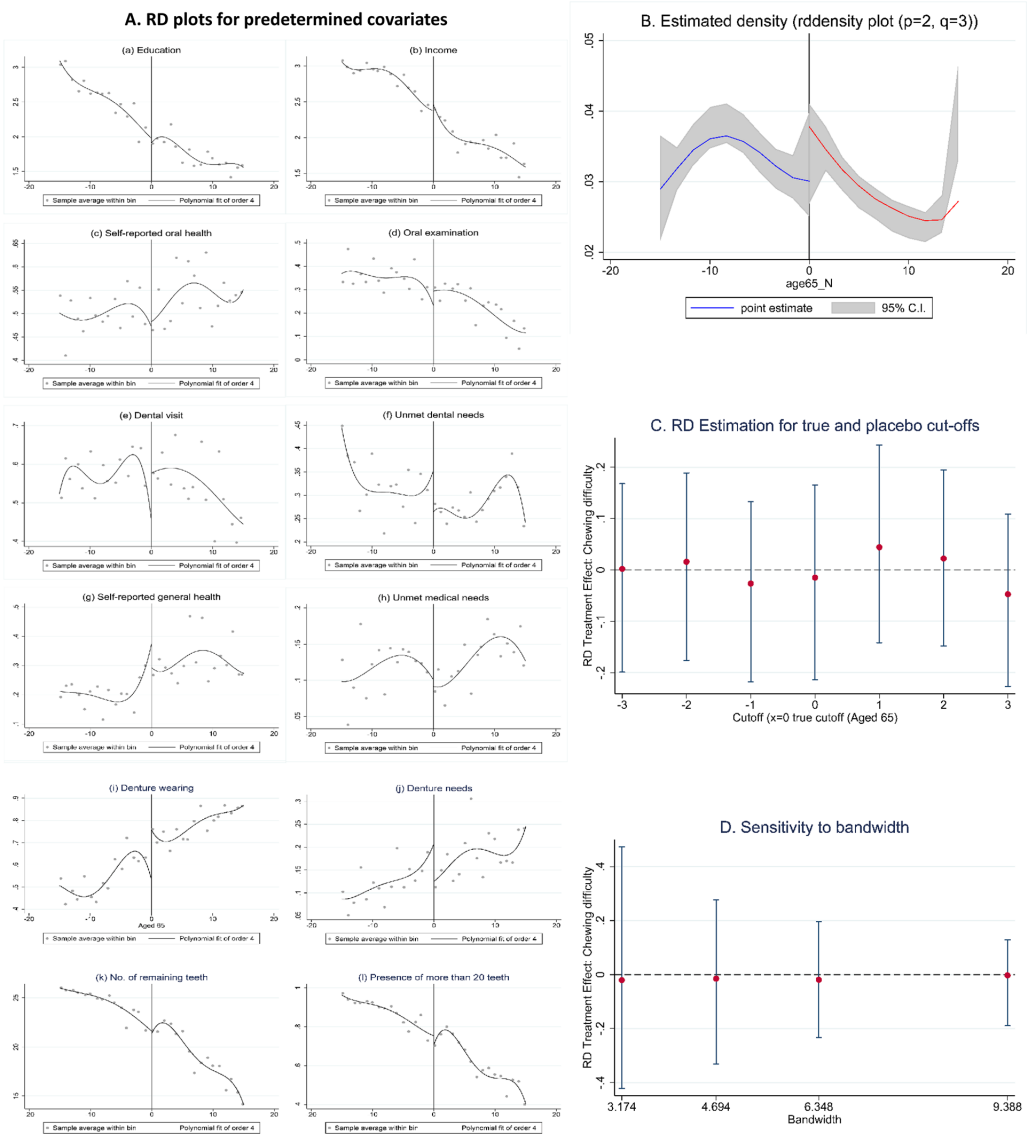
Validation and falsification of the running variable (aged 65) and outcome variable (chewing difficulty) for RD design: (**a**) RD plots for predetermined covariates, (**b**) Estimated density, (**c**) RD Estimation for true and placebo cut-offs, (**d**) Sensitivity to bandwidth.

Figure 2 shows (a) RD plot for predetermined covariates, (b) estimated density, (c) RD estimation for true and placebo cut-offs, and (d) sensitivity to bandwidth.

### Predetermined covariates

Our RD design appeared to be valid since we demonstrated that there were no discontinuities in any of the covariates (Fig. 2A). This graphical analysis within the optimal bandwidth showed that the right and left intercepts and the local linear fit were very close to each other in most cases. However, unmet dental needs (Fig. 2A–F), denture wearing (Fig. 2A–I) and denture needs (Fig. 2A–J) showed a more noticeable jump, though formal analysis indicated that this jump was not distinguishable from zero. All point estimates were small and 95% robust confidence intervals (CIs) included zero. In other words, there was no empirical evidence to suggest that these predetermined covariates were discontinuous at the cut-off (Supplementary Information Table S1).

### Density of running variable

The running variable density was valid for our RD design; Fig. 2B shows the actual density estimate with shaded 95% CIs. The density estimates for treated and control groups at the cut-off (the two intercepts in Fig. 2) were very close to each other, and the CIs (shaded areas) overlapped. The value of the statistic was − 0.774 with an associated P-value of 0.44. We failed to reject the null hypothesis that there would be no difference in the density between the two groups at the cut-off.

### Placebo cut-offs

The cut-off (aged 65 years) was suitable for our RD design. In Fig. 2C), the true cut-off of 0 is included as a reference for the comparison. All other cut-offs (± 3 years) are artificial or placebo, implying that treatment did not actually change at those points. We found that at all but one of the artificial cut-off points, the absolute value of the RD point estimate was smaller than the true RD estimate (− 0.015) and the P-values were above 0.3. Thus, chewing difficulty did not discontinuously vary at the artificial cut-offs (Supplementary Information Table S2).

### Sensitivity to bandwidth

The validity of our RD design was finally supported by changing the bandwidth used for local polynomial estimation. The results based on the CER-optimal choice (hCER = 3.174) were consistent with the results based on the MSE-optimal choice (hMSE = 4.694) and provided similar point estimates (Table 2). The two largest bandwidths (2·hCER = 6.348 and 2·hMSE = 9.388) provided results that were broadly consistent with the empirical findings obtained with the MSE-optimal selection.

The analysis showed (in Fig. 2D) that the bandwidth affected the results: as the bandwidth increased, the bias of the local polynomial estimator increased and its variance decreased; and as bandwidth increased, the confidence intervals decreased in length and were displaced due to bias.

## Discussion

This study evaluated whether the policy to improve dental insurance to cover dentures for those aged over 65 years led to an improvement in chewing ability in Korea using RD, a quasi-experimental design. Our null hypothesis was that there is no overall average treatment effect, namely, that there would be no discontinuity at the cut-off (roll-out of the new policy). In other words, non-eligible people would have similar rates of chewing ability as eligible people.

There were seemingly meaningful differences in the RD plot findings for the survey data when the periods before and after the policy change were compared. In 2010, there was no discontinuity at the cut-off, but in 2015 discontinuity could be observed in the plots. Additionally, after the expansion of dental insurance, chewing difficulties among those aged 65 years and above were 2.2–6.2% lower than the control (not eligible for insurance benefits). However, these results were not statistically significant in the RD regression analyses. It is unclear whether the discontinuity resulted from dental insurance coverage alone, since we did not find any statistically significant evidence for the treatment effect of potential outcomes in the RD design.

The group eligible for dental insurance benefits (aged 65 years and above) and the control group (aged under 65 years) may have different characteristics such as socioeconomic status, oral health behaviors, oral health status (receiving oral examination, dental visit, unmet dental needs, self-reported oral health, denture wear status, denture need, number of remaining teeth and presence of more than 20 teeth), and oral health-related factors (unmet medical needs, and self-reported general health)^2–5^. However, we controlled for these covariates in a robustness check with the same results.

The plausibility of our study design was supported by four falsification and sensitivity tests. We determined that there were no discontinuities for the predetermined covariates, meaning that the covariates were not affected by denture insurance benefits (Fig. 2A, Supplementary Information Table S1). If the covariates (e.g. income, dental visit, self-reported oral health, denture status, and the number of teeth) that are strongly correlated with chewing difficulty are discontinuous at ‘aged 65’, the potential outcome functions are unlikely to show continuity^27–29^.

Second, we validated the density of the running variable (age). We tested the null hypothesis that the density of the running variable would be continuous at the cutoff. As a result, there was no difference in the density between the two groups at the cut-off. Since we did not find statistically significant evidence of discontinuity at the cut-off, we failed to reject the null hypothesis, which offers evidence supporting the validity of the RD design (Fig. 2B).

Third, the validity of our cut-off was proven because chewing difficulty did not discontinuously jump when artificial cut-offs of ages 62 and 68 were considered. Testing the age of 65 cut-offs is important because of the possibility of inaccurate reporting of age in the survey. Thus, artificial cut-offs ranging from 62 to 68 years were used for comparison (Fig. 2C).

Finally, the sensitivity test confirmed that bandwidth affected the results. The results based on the CER-optimal choice were consistent with the results based on the MSE-optimal choice and gave similar point estimates. The two largest bandwidths led to results that were broadly consistent with the empirical findings obtained with the MSE-optimal choice (Fig. 2D).

The null results of the study have two implications for future studies. First, the impact of the policy on chewing ability may not be evident within the time frame considered. For example, many eligible individuals may not have been aware of the policy change, or even if they were aware, they may not have the time to take up the offer of expanded denture coverage benefits. Second, the impact of denture insurance should be evaluated using an expanded set of oral health indicators besides chewing difficulty. Thus, future studies are required to assess the full impact of dental insurance in Korea. The quality and extent of insurance benefits are expected to improve. Cross-sectional and longitudinal data spanning four to ten years have suggested that Medicare eligibility for those over the age of 65 years is associated with a reduction in sociodemographic inequalities, general health problems, and mortality^25,30,31^. However, there is currently limited evidence that expanded dental insurance benefits lead to a reduction of oral health issues in Korea.

Oral examinations are free nationwide, and the cost of dental visits for those over the age of 65 years is usually low (an individual aged over 65 years could pay one-tenth of the visit cost, approximately 1.28 USD, that an individual under the age of 65 years would pay). However, in the present study there were no differences in oral examinations and dental visits between the groups studied (Table 1, Fig. 2A–D and A–E). One of the reasons we found that chewing difficulties did not significantly improve in those over the age of 65 years with denture insurance benefits was because utilization of dental services such as dental examinations has not improved.

It is necessary to consider indicators that assess social well-being outcomes in addition to oral health. Chewing difficulty is a very subjective indicator^2,15^. It not only depends on the intraoral status (dental caries, periodontal disease, number of teeth, prosthodontics, dentures, and/or dry mouth, etc.), but also on psycho-social factors (food preferences, treatment information, expectations, and trust, etc.) which are affected by broader considerations^4,32,33^. For example, chewing ability after denture treatments is associated with the perception of dental specialties, the treating dentists, and dental hospitals^34,35^. Therefore, improvement in chewing ability is not the only benefit of dentures^15^. Previous reports have suggested that dental issues affect both oral health and social outcomes^36–39^. Recent oral health survey tools implemented by the WHO include an assessment of speech problems (or trouble pronouncing words), embarrassment due to the appearance of teeth, and reduced participation in social activities^26^.

Studies evaluating the impact of denture insurance should also assess indicators for enhanced nutrition, overall health, sustained cognitive function^3–5^, decreased social burdens (for example saving on insurance costs), recovery of self-esteem, appearance, maintenance of social activities, and quality of life and happiness due to oral health interventions.

Importantly, our RD design had some limitations. First, we evaluated only the relatively short-term impact of the policy change. Second, our analysis may have residual bias due to differences in unobserved covariates^17^, because we only used predetermined covariates available from the KNHANES data.

Nevertheless, we used a quasi‐experimental design to assess causal inference between the expansion of dental health insurance and improvement in chewing ability (i.e. bias from unmeasured confounding) by assessing the impact of differences in coverage eligibility that were plausibly exogenous, for all observed or unobserved predictors.

In conclusion, including denture treatment in the dental insurance scheme for the older adults could improve chewing ability outcomes after sustained implementation of the policy. However, long-term evaluations should not only consider oral health indicators (such as chewing ability), but also social well-being outcomes (aesthetic appearance, speech, social relationships, and life satisfaction).

## Supplementary information

Supplementary Information.

## Data Availability

The data from the fourth KNHANES is open to the public, therefore, any researcher can obtain data upon request from https://knhanes.cdc.go.kr. The Korean National Health and Nutrition Examination Survey (KNHANES) data are publicly available through the KNHANES website (https://knhanes.cdc.go.kr/knhanes/eng/sub03/sub03_01.do).
