# Supplementary material for: Did expanded access to denture services improve chewing ability in the Korean older population? Results of a regression discontinuity analysis
Source: Sci Rep. 2020 Jul 17;10:11859. doi: 10.1038/s41598-020-68189-7 (PMC7368076; doi:10.1038/s41598-020-68189-7)
Supplement: Supplementary file 1 — Supplementary Information. [file 41598_2020_68189_MOESM1_ESM.docx]

**Supplementary information**

**Did expanded access to denture services improve chewing ability in the Korean older population? Results of a regression discontinuity analysis**

*Nam-Hee Kim^1,2^ & Ichiro Kawachi^2^

^1^Department of Dental Hygiene, Wonju College of Medicine, Yonsei University, Wonju, South Korea, nami71@yonsei.ac.kr, nhkim@hsph.harvard.edu

^2^Department of Social and Behavioral Sciences, Harvard T.H. Chan School of Public Health, Boston, Massachusetts, ikawachi@hsph.harvard.edu

***Corresponding Author:**

N.H. Kim, MPH, PhD

Department of Dental Hygiene, Wonju College of Medicine, Yonsei University, 20 Ilsan-ro, Wonju, Gangwon-do 26426, Korea

Telephone: +82-33-741-0391

Fax: +82-33-735-0391

E-mail: [nami71@yonsei.ac.kr](mailto:nami71@yonsei.ac.kr)

ORCID 0000-0001-5463-0073

**Table S1. Continuity-Based Analysis for Predetermined Covariates**

| Variable | MSE-Optimal Bandwidth | RD Estimator | Robust Inference | | | Effect; Number of Observations | |
| --- | --- | --- | --- | --- | --- | --- | --- |
|  |  |  | P-value | CI | | Control | Treated |
| Gender | 5.343 | 0.047 | 0.469 | -0.115 | 0.249 | 427 | 483 |
| Education | 5.844 | -0.010 | 0.942 | -0.390 | 0.362 | 427 | 483 |
| Income | 5.482 | 0.112 | 0.404 | -0.216 | 0.535 | 427 | 483 |
| Spouse | 5.468 | -0.026 | 0.684 | -0.174 | 0.114 | 427 | 483 |
| Oral health | 5.403 | 0.000 | 0.873 | -0.167 | 0.196 | 427 | 483 |
| Oral examination | 5.894 | 0.006 | 0.835 | -0.142 | 0.176 | 427 | 483 |
| Dental visit | 3.511 | 0.089 | 0.297 | -0.113 | 0.371 | 250 | 345 |
| Unmet dental needs | 5.967 | -0.052 | 0.530 | -0.211 | 0.108 | 427 | 483 |
| General health | 3.994 | -0.093 | 0.240 | -0.325 | 0.081 | 250 | 345 |
| Unmet medical needs | 5.390 | -0.015 | 0.934 | -0.117 | 0.107 | 427 | 483 |
| Denture wearing | 4.169 | 0.126 | 0.154 | -0.057 | 0.363 | 329 | 416 |
| Need for denture | 4.490 | -0.776 | 0.220 | -0.255 | 0.058 | 329 | 416 |
| No. of present teeth | 3.619 | 1.040 | 0.341 | -1.719 | 4.972 | 427 | 320 |
| Having more than 20 teeth | 3.357 | 0.522 | 0.334 | -0.105 | 0.310 | 427 | 320 |
| Total number of observations = 2,513  Sharp RD estimates using local polynomial regression  CI, confidence interval | | | | | | | |

| **Table S2. Continuity-Based Analysis for Placebo Cut-offs** | | | | | | | |
| --- | --- | --- | --- | --- | --- | --- | --- |
| Alternative Cut-offs | MSE-Optimal Bandwidth | RD Estimator | Robust Inference | | | Effect; Number of Observations | |
|  |  |  | P-value | CI | | Control | Treated |
| -3 | 4.650 | 0.002 | 0.868 | -0.199 | 0.168 | 370 | 408 |
| -2 | 4.865 | 0.016 | 0.946 | -0.176 | 0.189 | 352 | 421 |
| -1 | 5.131 | -0.027 | 0.633 | -0.218 | 0.133 | 433 | 506 |
| **0** | **4.694** | **-0.015** | **0.803** | **-0.214** | **0.166** | **329** | **416** |
| 1 | 4.966 | 0.044 | 0.605 | -0.142 | 0.243 | 321 | 412 |
| 2 | 5.668 | 0.022 | 0.790 | -0.148 | 0.194 | 408 | 448 |
| 3 | 5.625 | -0.047 | 0.490 | -0.227 | 0.109 | 421 | 438 |
| Total number of observations = 2,513  Sharp RD estimates using local polynomial regression  CI is confidence interval | | | | | | | |
